# Metropolitan Hospital Sunday Fund

**Published:** 1903-08-15

**Authors:** 


### HOSPITAL ADMINISTRATION.

### CONSTRUCTION AND ECONOMICS.

### METROPOLITAN HOSPITAL SUNDAY FUND.

(*Continued from Page* 336.)

### II.—THE AWARDS IN DETAIL.

It cannot fail to be of interest in continuation of our article last week to proceed to show in detail what each of the majority of the institutions received from the Hospital Sunday Fund this year as compared with last year's grant. It must further be a matter of great interest, not only to the London hospital managers, but to the managers of all hospitals throughout the country, to compare the approximate cost per head, as stated by the hospital authorities—that is, the cost per in-patient per week during 1902. It will be observed how great is the difference which actually exists between hospitals doing the same kind of work under similar conditions, whether these hospitals have medical schools attached to them or not; whether they are situated in the centre of a crowded population or are doing their work quietly in some country district in the neighbourhood of a great city. It will be seen that in every class of institution the differences shown in this matter of expenditure per in-patient per week are remarkable, and we quite agree with the Distribution Committee in thinking that, when the cost per occupied bed per week in 1902 varies from £1 1s. 3d. to £2 18s. 5d. in twenty-one of the principal hospitals, such an expenditure is higher than is justified by a due allowance for the necessary increase which the progress of scientific treatment and nursing has caused.

#### TABLE B.—GENERAL HOSPITALS.

*The following table shows the number of beds, the cost per in-patient per week as worked out by the hospital authorities, the awards to each hospital or institution in* 1902 *and* 1903, *and the increase or decrease of such awards.*

| Name of Institution. | No. of Beds. | In-patient Cost per Week.* | Awards. | | Decrease or Increase. |
|---|---|---|---|---|---|
| | | | 1902 | 1903. | |
| General Hospitals. | | £ s. d. | £ | £ | £ |
| St. George's.. .. .. | 351 | 2 14 10 | 1,917 | 1,137 | − 780 |
| Westminster .. .. | 213 | 2 4 9 | 1,390 | 1,452 | + 62 |
| Hampstead .. .. .. | 32 | 2 4 3 | 240 | 227 | − 13 |
| University College .. | 188 | 2 2 7 | 1.303 | 1.798 | + 495 |
| King's College .. .. | 223 | 2 1 10 | 1,437 | 1 527 | + 90 |
| Middlesex .. .. .. | 296 | 2 1 2 | 2,310 | 2,416 | + 431 |
| ,, Cancer Wing, new extra grant .. | 45 | 2 5 1 | No grant | 325 | |
| Guy's .. .. .. | 652 | 2 0 5 | 2 875 | 2,275 | − 600 |
| London .. .. .. | 790 | 2 0 0 | 6.181 | 5 547 | − 634 |
| Charing Cross .. .. | 150 | 1 19 3 | 1,207 | 1 083 | − 124 |
| Poplar .. .. .. | 103 | 1 19 1 | 623 | 617 | − 6 |
| St. Thomas' .. .. | 586 | 1 18 10 | 1 342 | 292 | − 1,050 |
| Metropolitan .. .. | 111 | 1 17 7 | 862 | 953 | + 91 |
| St. Mary's .. .. .. | 281 | 1 17 2 | 2 492 | 2,600 | + 108 |
| Great Northern Central .. | 159 | 1 11 2 | 1,054 | 1,121 | + 67 |
| North-West London .. | 53 | 1 10 8 | 431 | 477 | + 46 |
| "Dreadnought" Seamen's | 269 | 1 10 3 | 1,294 | 1 592 | + 298 |
| West Ham .. .. .. | 60 | 1 9 9 | 527 | 563 | + 36 |
| Mildmay Mission .. .. | 50 | 1 8 7 | 287 | 314 | + 27 |
| Royal Free .. .. .. | 170 | 1 7 11 | 1,246 | 1,300 | + 54 |
| French Hospital .. .. | 70 | 1 5 10 | 307 | 347 | + 40 |
| West London .. .. | 146 | 1 5 8 | 815 | 1,170 | + 355 |
| German .. .. .. | 130 | 1 4 8 | 594 | 617 | + 23 |
| St. John and St. Elizabeth | 110 | 1 4 5 | 144 | 276 | + 132 |
| Tottenham .. .. .. | 73 | 1 3 7 | 383 | 390 | + 7 |

* As stated by hospital authorities, Sunday Fund authorities make the cost in many cases much higher.

In considering the figures in the preceding and following tables it must be understood that we have not given every institution to which an award has been granted because some of them appear to us to have no special interest, or are too small to be of sufficient importance to enumerate. We have also omitted the dispensaries, feeling that the total awards made, as given in the foregoing table, will supply all the information which it is necessary to publish for our present purposes.

#### TABLE C.—CONSUMPTION HOSPITALS.

*The following table shows the number of beds, the cost per in-patient per week as worked out by the hospital authorities, the awards to each hospital or institution in* 1902 *and* 1903, *and the increase or decrease of such awards.*

| Name of Institution. | No. of Beds. | In-patient Cost per Week.* | Awards. | | Decrease or Increase. |
|---|---|---|---|---|---|
| | | | 1902. | 1903. | |
| Consumption Hospitals. | | £ s. d. | £ | £ | £ |
| Brompton Consumption.. | 315 | 2 4 3 | 1,821 | 1,733 | − 88 |
| Royal Chest .. .. | 80 | 1 18 4 | 383 | 487 | + 104 |
| Royal National Ventnor.. | 154 | 1 18 4 | 383 | 379 | − 4 |
| Mount Vernon, Hampstead .. .. .. | 100 | 1 13 10 | 671 | 780 | + 109 |
| City of London, Victoria Park .. .. .. | 164 | 1 10 10 | 958 | 1,105 | + 147 |

* As stated by hospital authorities, Sunday Fund authorities make the cost in many cases much higher.

Hospitals for consumption, if properly administered on modern principles, should, in our judgment, be capable of showing the minimum cost at which a well-regulated hospital can maintain a patient during the period of severe illness. The cost of the nursing and even the cost of many of the articles of diet and of the supplies included in the treatment are on the whole relatively inexpensive, and we feel very strongly that the Brompton Hospital and one or two others in the group are spending far too much per in-patient per week at the present time, and that the managers will be well advised to direct their attention closely to the subject with a view to a large reduction in the present expenditure as set forth in the above table.

The following table indeed affords much food for reflection to the thoughtful. When one of the very best managed hospitals for children in the country, a hospital which it is always a pleasure to visit, and which reveals at all hours of the day and night the most careful and thorough system of administration, can maintain the highest state of efficiency at a cost of £1 3s. 2d. per child per week, we feel very strongly that other institutions, where the expenditure amounts to 50 or 75 per cent. more than the

### TABLE D.—CHILDREN'S HOSPITALS.

*The following table shows the number of beds, the cost per in-patient per week as worked out by the hospital authorities, the awards to each hospital or institution in* 1902 *and* 1903, *and the increase or decrease of such awards.*

| Name of Institution. | No. of Beds. | In-patient Cost per Week.* | Awards. | | Decrease or Increase. |
|---|---|---|---|---|---|
| | | | 1902. | 1903. | |
| Children's Hospitals. | | £ s. d. | £ | £ | £ |
| Evelina .. .. .. | 66 | 2 18 6 | 287 | 217 | − 70 |
| Victoria .. .. .. | 92 | 1 18 0 | 460 | 477 | + 17 |
| North-Eastern .. .. | 57 | 1 16 3 | 287 | 390 | + 103 |
| Paddington Green .. | 46 | 1 15 0 | 297 | 298 | + 1 |
| Hospital for Sick Children | 252 | †1 13 5 | 1,054 | 975 | − 79 |
| Belgrave .. .. .. | 26 | 1 6 7 | 125 | 114 | − 11 |
| St. Mary's, Plaistow .. | 35 | 1 5 11 | 249 | 282 | + 33 |
| Alexandra .. .. .. | 76 | 1 3 6 | 240 | 303 | + 63 |
| East London .. .. | 135 | 1 3 2 | 767 | 932 | + 165 |
| St. Monica's Home .. | 28 | 1 0 5 | 96 | 87 | − 9 |
| Cheyne .. .. .. | 50 | 1 0 1 | 125 | 119 | − 6 |
| Sevenoaks .. .. .. | 34 | 0 17 2 | 43 | 49 | + 6 |

* As stated by hospital authorities, Sunday Fund authorities make the cost in many cases much higher.
† Including patients in convalescent home.

sum named, should put their house in order without delay. The figures for the Hospital for Sick Children are not comparable with those of the other hospitals in the table for the reason that the calculation has been made upon figures which include the patients in the convalescent home, and so, we presume, the cost per in-patient per week as returned is smaller than it otherwise would be. It will be noted that probably the most efficient of all the hospitals included in the table has received a grant of £165 more in 1903 than it received in 1902, and that another hospital has had its grant reduced by £70 this year, the cost of each of its in-patients per week being more than 100 per cent. higher, and is indeed so high as to be unaccountable, except it may be due to some exceptional circumstance, such as the closing of most of the beds in the hospital during the greater portion of the year. We may remark that the three hospitals last mentioned in the table are practically confined to chronic or incurable cases, which accounts no doubt for the small cost per in-patient.

### TABLE E.—LYING-IN HOSPITALS.

*The following table shows the number of beds, the cost per in-patient per week as worked out by the hospital authorities, the awards to each hospital or institution in* 1902 *and* 1903, *and the increase or decrease of such awards.*

| Name of Institution. | No. of Beds. | In-patient Cost per Week.* | Awards. | | Decrease or Increase. |
|---|---|---|---|---|---|
| | | | 1902. | 1903. | |
| Lying in Hospitals. | | £ s. d. | £ | £ | £ |
| British .. .. .. | 20 | 2 12 0 | 29 | 16 | − 13 |
| General .. .. .. | 24 | 2 2 8 | 57 | 32 | − 25 |
| Clapham Maternity .. | 30 | 1 18 0 | 38 | 22 | − 16 |
| East End Mothers' Home.. | 16 | 1 17 10 | 77 | 76 | − 1 |
| Queen Charlotte's .. .. | 74 | 1 15 8 | 479 | 477 | − 2 |
| City of London .. .. | 42 | 1 13 8 | Nil | 100 | + 100 |

* As stated by hospital authorities, Sunday Fund authorities make the cost in many cases much higher.

This group shows considerable differences in the cost per in-patient, and the same remarks which we have made already in regard to other institutions apply with equal force, and will, we hope, receive due attention at the hands of the committees immediately concerned.

### TABLE F.—HOSPITALS FOR WOMEN.

*The following table shows the number of beds, the cost per in-patient per week as worked out by the hospital authorities, the awards to each hospital or institution in* 1902 *and* 1903, *and the increase or decrease of such awards.*

| Name of Institution. | No. of Beds. | In-patient Cost per Week.* | Awards. | | Decrease or Increase. |
|---|---|---|---|---|---|
| | | | 1902. | 1903. | |
| Hospitals for Women. | | £ s. d. | £ | £ | £ |
| Hospital for Women .. | 46 | 2 8 9 | 144 | 130 | − 14 |
| Samaritan Free .. .. | 47 | 2 7 10 | 492 | 439 | − 53 |
| Chelsea .. .. .. | 52 | 2 2 7 | 422 | 379 | − 43 |
| Grosvenor .. .. .. | 23 | 2 0 5 | 172 | 162 | − 10 |
| New .. .. .. .. | 52 | 1 19 9 | 307 | 325 | + 18 |
| Royal for Children and Women .. .. .. | No return | No return | 268 | 217 | − 51 |

* As stated by hospital authorities, Sunday Fund authorities make the cost in many cases much higher.

It will be seen that the hospitals for women expend on an average considerably more per in-patient per week than most of the other hospitals. We should be glad to have some explanation of the causes which lead to this expenditure, and it will be very interesting to publish them for the information of all who are interested in the subject.

### TABLE G.—OTHER SPECIAL HOSPITALS.

*The following table shows the number of beds, the cost per in-patient per week as worked out by the hospital authorities, the awards to each hospital or institution in* 1902 *and* 1903, *and the increase or decrease of such awards.*

| Name of Institution. | No. of Beds. | In-patient Cost per Week.* | Awards. | | Decrease or Increase. |
|---|---|---|---|---|---|
| | | | 1902. | 1903. | |
| Other Special Hospitals. | | £ s. d. | £ | £ | £ |
| London Fever .. .. | 200 | 3 14 8 | 719 | 758 | + 39 |
| St. Peter's, for Stone .. | 27 | 2 13 2 | 96 | 92 | − 4 |
| West End, for Nervous Diseases .. .. .. | 50 | 2 9 10 | 240 | 217 | − 23 |
| St. Mark's, for Fistula .. | 37 | 2 6 10 | 144 | 130 | − 14 |
| Middlesex Cancer Wing .. | 45 | 2 5 1 | Nil | 325 | + 325 |
| Royal London Ophthalmic | 138 | 2 3 10 | 862 | 942 | + 80 |
| Cancer .. .. .. | 105 | 2 3 4 | 527 | 325 | − 202 |
| Hospital for the Throat .. | 33 | 2 1 1 | 249 | 108 | − 141 |
| National, for Heart .. | 25 | 1 18 6 | 77 | 87 | + 10 |
| Gordon, for Fistula .. | 27 | 1 18 0 | 24 | 22 | − 2 |
| National, for Paralysed .. | 196 | 1 17 5 | 958 | 1148 | + 190 |
| Central London Throat and Ear .. .. .. | 17 | 1 14 1 | 48 | 54 | + 6 |
| London Throat .. .. | 16 | 1 9 5 | 29 | 32 | + 3 |
| Metropolitan Ear, Nose, and Throat .. .. | 17 | 1 8 11 | 29 | Nil | − 29 |
| Royal Westminster Ophthalmic .. .. .. | 40 | 1 8 9 | 134 | 184 | + 50 |
| St. John's, for Skin .. | 50 | 1 7 0 | 57 | 54 | − 3 |
| Royal Orthopædic.. .. | 50 | 1 5 3 | 134 | 81 | − 53 |
| Central London Ophthalmic .. .. .. | 28 | 1 5 1 | 115 | 184 | + 69 |
| Royal Sea-Bathing, Margate .. .. .. | 160 | 1 4 3 | 287 | 325 | + 38 |
| Royal Ear .. .. .. | 9 | 1 3 1 | 19 | 11 | − 8 |
| Western Ophthalmic .. | 13 | 1 2 9 | 48 | 54 | + 6 |
| Female Lock .. .. | 135 | 1 0 6 | 307 | 227 | − 80 |
| National Orthopædic .. | 60 | 18 9 | 125 | 179 | + 54 |
| Royal Eye .. .. .. | 42 | 18 8 | 240 | 314 | + 74 |
| City Orthopædic .. .. | 45 | 15 1 | 249 | 233 | − 16 |

* As stated by hospital authorities, Sunday Fund authorities make the cost in many cases much higher.

Of course, everybody familiar with hospital work will realise that the position of the London Fever Hospital is peculiar from the circumstance that the cost per bed each year must largely depend on whether there has been any epidemic disease during the period to which the figures relate. When scarlet fever is rampant and the hospital is full the cost per in-patient per week comes down to a normal level; when there is no epidemic and relatively few beds are occupied the cost per in-patient in this class of hospital must necessarily be high. It will be observed that the seven hospitals which immediately follow the London Fever Hospital in the table show each a cost per in-patient per week exceeding £2. We merely state the fact and call attention to it because we should be glad to have any explanation of the causes which have led to this expenditure, so that justice may be done in each case to the management. Of the remaining hospitals in the table it may be said that a difference which varies from a weekly cost per in-patient of £1 18s. 6d. to one of 15s. 1d. presents features for inquiry and explanation which we have little doubt will be forthcoming without delay.

### TABLE H.—CONVALESCENT INSTITUTIONS.

*The following table shows the number of beds, the cost per in-patient per week as worked out by the hospital authorities, the awards to each hospital or institution in* 1902 *and* 1903, *and the increase or decrease of such awards.*

| Name of Institution. | No. of Beds. | In-patient Cost Per Week.* | Awards. | | Decrease or Increase. |
|---|---|---|---|---|---|
| | | | 1902. | 1903 | |
| Convalescent Institutions. | | £ s. d. | £ | £ | £ |
| Mary Wardell, for Scarlet Fever .. .. .. | 40 | 3 5 0 | 105 | 97 | — 8 |
| Middlesex, E. Clacton .. | 51 | 1 19 10 | — | — | — |
| King's College, Hemel Hempstead .. .. | 40 | 1 11 0 | 172 | 70 | — 102 |
| Charing Cross, Limpsfield | 50 | 1 1 7 | 86 | 100 | + 14 |
| Chelsea, for Women, St. Leonard's .. .. | 22 | 14 0 | 48 | 43 | — 5 |
| Homœopathic, Eastbourne | 18 | 1 8 0 | 19 | 22 | + 3 |
| Metropolitan, Cranbrook | 376 | 13 4 | 671 | 704 | + 33 |
| Bexhill .. .. .. | 165 | 15 6 | 383 | 379 | — 4 |
| All Saints', Eastbourne .. | 340 | 12 3 | 383 | 433 | + 50 |
| St. Andrew's, Folkestone | 130 | 14 8 | 240 | 195 | — 45 |
| Morley House, for Working men, St. Margaret's Bay .. .. .. | 120 | 18 10 | 211 | 190 | — 21 |
| Seaside Convalescent, Seaford .. .. .. | 110 | 19 6 | 96 | 108 | + 12 |
| Friendly Societies', Dover | 100 | 18 2 | 38 | 76 | + 38 |
| Mrs. Gladstone's, Mitcham | 60 | 12 10 | 48 | 65 | + 13 |

* As stated by hospital authorities, Sunday Fund authorities make the cost in many cases much higher.

This table is interesting because the first institution mentioned is the only convalescent home which takes in scarlet-fever cases, and here the cost per week is extraordinarily high, namely, £3 5s. per in-patient. The next five institutions have a special interest because they represent a new departure on the part of certain London general hospitals which have the good fortune to possess branch hospitals in the country to which they can send suitable patients before their wounds are healed. This fact alone may properly account for the higher expenditure which the figures show in most cases; but it would be interesting to have information as to the causes which account for so large a difference as that between the cost of £1 19s. 10d. per in-patient per week at Clacton and of £1 1s. 7d. per week per in-patient at Limpsfield. The remaining convalescent institutions of the old type seem to be administered, on the whole, on the same general principles, if the management may be judged by the cost of each in-patient per week. It is always supposed that an institution of this class situated at the seaside entails a larger expenditure than one situated in the country. Why we have never been able to understand; but now the point is raised, possibly some explanation may be forthcoming through the courtesy of the managers of some, at any rate, of these, institutions.

### TABLE I.—COTTAGE HOSPITALS AND MISCELLANEOUS INSTITUTIONS.

*The following table shows the number of beds, the cost per in-patient per week as worked out by the hospital authorities the awards to each hospital or institution in* 1902 *and* 1903, *and the increase or decrease of such awards.*

| Name of Institution. | No. of Beds. | In-patient Cost per Week.* | Awards. | | Decrease or Increase. |
|---|---|---|---|---|---|
| | | | 1902. | 1903. | |
| Cottage Hospitals and Miscellaneous Institutions. | | £ s. d. | £ | £ | £ |
| Establishment for Gentlewomen .. .. .. | 20 | 3 9 7 | 105 | 119 | + 14 |
| St. Saviour's Nursing Home .. .. .. | 27 | 2 9 2 | 115 | 130 | + 15 |
| Epsom and Ewell Cottage | 16 | 2 8 4 | 62 | 70 | + 8 |
| Bolingbroke .. .. | 30 | 2 4 10 | Nil | 249 | + 249 |
| St. John's Lewisham .. | 41 | 1 17 11 | 96 | 162 | + 66 |
| Bushey Heath Cottage .. | 20 | 1 18 0 | 48 | 49 | + 1 |
| Friedenheim, for the Dying | 42 | 1 17 9 | 287 | 325 | + 38 |
| Passmore Edwards Cottage, Tilbury .. .. | 12 | 1 17 6 | 38 | 32 | — 6 |
| Sidcup Cottage .. .. | 14 | 1 17 4 | 38 | 32 | — 6 |
| Acton Cottage .. .. | 12 | 1 15 9 | Nil | 76 | + 76 |
| Livingstone Cottage, Dartford .. .. .. | 18 | 1 13 0 | 86 | 97 | + 11 |
| Beckenham Cottage .. | 30 | 1 12 9 | 67 | 87 | + 20 |
| Blackheath and Charlton Cottage .. .. .. | 18 | 1 11 2 | 77 | 87 | + 10 |
| Bromley Cottage .. .. | 32 | 1 10 6 | 105 | 130 | + 25 |
| Canning Town Cottage .. | 13 | 1 9 11 | 48 | 54 | + 6 |
| Memorial Cottage, Mildmay Park .. .. | 30 | 1 8 6 | 38 | 32 | — 6 |
| Eltham Cottage .. .. | 11 | 1 7 10 | 48 | 65 | + 17 |
| Wimbledon Cottage .. | 16 | 1 6 11 | 53 | 65 | + 12 |
| Passmore Edwards Cottage, Willesden .. .. | 25 | 1 5 9 | 86 | 141 | + 55 |
| Reigate and Redhill Cottage .. .. .. | 30 | 1 3 6 | 86 | 125 | + 39 |
| Woolwich and Plumstead Cottage .. .. .. | 10 | 1 3 6 | 43 | 60 | + 17 |
| Enfield Cottage .. .. | 14 | 1 3 2 | 57 | 76 | + 19 |
| Hounslow Cottage .. | 15 | 1 3 1 | 29 | 49 | + 20 |
| Chislehurst Cottage .. | 21 | 1 0 11 | 57 | 87 | + 30 |

* As stated by hospital authorities, Sunday Fund authorities make the cost in many cases much higher.

Possibly this table, which is the last, will excite as much general interest as any one we have given. There is a very wide and extended interest in cottage hospitals, and the awards reveal differences which cannot fail to create comment and lead to inquiry. The first two institutions in the list are special, but it will be noticed that there is a difference of £1 per week in the cost of each in-patient, which we are unable to explain, seeing that the work done in each of the two institutions is practically identical. We should very much like to know why it is that an in-patient in the Epsom Cottage Hospital costs £2 8s. 4d. per week when the authorities at Chislehurst Cottage Hospital, which has only five more beds, can do the same work for £1 0s. 11d. per week. We conclude that the awards made in the various cases may be taken as indicating pretty definitely the opinion of the Distribution Committee of the Sunday Fund in regard, not only to the merits and needs, but to the management and expenditure of each hospital and institution included in our tables.

